# Application of Methyl Jasmonate to Papaya Fruit Stored at Lower Temperature Attenuates Chilling Injury and Enhances the Antioxidant System to Maintain Quality

**DOI:** 10.3390/foods12142743

**Published:** 2023-07-19

**Authors:** Jianhui Li, Muhammad Azam, Amtal Noreen, Muhammad Ali Umer, Riadh Ilahy, Muhammad Tahir Akram, Rashad Qadri, Muhammad Arslan Khan, Shoaib ur Rehman, Imtiaz Hussain, Qiong Lin, Hongru Liu

**Affiliations:** 1 College of Chemistry and Materials Engineering, Quzhou University, Quzhou 324000, China; lijianhui@yeah.net; 2 Pomology Laboratory, Institute of Horticultural Sciences, Faculty of Agriculture, University of Agriculture, Faisalabad 38040, Pakistan; amtalnoreen@gmail.com (A.N.); maliumer1188@gmail.com (M.A.U.); rashad.qadri@uaf.edu.pk (R.Q.); marslanuaf@gmail.com (M.A.K.); 3 Laboratory of Horticulture, National Agricultural Research Institute of Tunisia (INRAT), University of Carthage, Ariana 1054, Tunisia; bn.riadh@gmail.com; 4 Department of Horticulture, PMAS-Arid Agriculture University, Rawalpindi 46300, Pakistan; tahiruaf786@gmail.com; 5 Department of Horticulture, University of Agriculture, Faisalabad, Sub Campus Depalpur, Okara 53600, Pakistan; shoaib.rehman@uaf.edu.pk; 6 Winrock International, Sindh 71000, Pakistan; imtiazhort@gmail.com; 7 Institute of Food Science and Technology, Chinese Academy of Agricultural Science, Beijing 100081, China; linqiong@caas.cn; 8 Institute of Crop Breeding & Cultivation Research, Shanghai Academy of Agricultural Sciences, Shanghai 201403, China

**Keywords:** MeJA application, papaya quality deterioration, antioxidant activities, reactive oxygen species, sensory traits, extended cold storge

## Abstract

Papaya fruit has a limited shelf life due to its sensitivity to decay and chilling damage during cold storage. The application of methyl jasmonate (MeJA) is known to reduce the incidence of disease and chilling injury, and to maintain the overall quality of the papaya fruit when stored at low temperature. Consequently, the effects of postharvest MeJA (1 mM) immersion on papaya fruits during low-temperature storage (10 °C ± 2 °C) for 28 days were studied. The experiment revealed that MeJA treatment significantly decreased the papaya fruit’s weight loss, disease incidence, and chilling injury index. Furthermore, the accumulation of malondialdehyde and hydrogen peroxide was markedly lower after the application of MeJA. In addition, MeJA treatment exhibited significantly higher total phenols, ascorbic acid, antioxidant activity, and titratable acidity in contrast to the control. Similarly, MeJA-treated papaya fruits showed higher antioxidant enzymatic activity (superoxide dismutase, catalase, and peroxidase enzymes) with respect to the control fruits. In addition, MeJA reduced the soluble solids content, ripening index, pH, and sugar contents compared to the control fruits. Furthermore, MeJA-treated papaya fruit exhibited higher sensory and organoleptic quality attributes with respect to untreated papaya fruits. These findings suggested that postharvest MeJA application might be a useful approach for attenuating disease incidence and preventing chilling injury by enhancing antioxidant activities along with enhanced overall quality of papaya fruits during low-temperature storage.

## 1. Introduction

Papaya (*Carica papaya* L.) is a high-quality, delicious berry fruit and is famous for its outstanding nutritional composition and health-promoting properties. However, limited shelf life, massive quality losses, and susceptibility to postharvest diseases during storage are the major challenges facing the papaya industry worldwide [1]. Papaya fruits synthesizes a substantial level of ethylene to stimulate ripening, like most climacteric fruits. The higher respiration rate and climacteric ripening pattern of papaya fruit promote pulp softening and several physicochemical changes that limit its long-term storage [2]. Additionally, the drastic deterioration of fresh papaya quality due to fungal diseases throughout the papaya storage and transportation chain results in huge economic losses to the papaya sector [3]. Gajanana et al. [4] demonstrated that postharvest papaya losses from field to consumer level ranged between 25 and 60%, depending upon supply chains management operations. Low-temperature storage has been recognized as the most effective strategy for extending fresh produce’s shelf life and reducing quality losses [5]. The optimal temperature during storage depends on the development stage of the papaya fruit. Generally, 13 °C temperature is recommended for the mature green to one-fourth yellow papayas, while for partially ripe papaya fruits (one-fourth to one-half yellow) the recommended temperature is 10 °C. However, 7 °C is considered the optimal storage temperature for ripe papayas [6]. However, papaya is quite affected by storage at low temperature, as highlighted by chilling injuries. Moreover, the occurrence of chilling symptoms under low-temperature storage is another important limiting factor for papaya fruit quality below 10 °C [7]. The typical signs of chilling injury involve sophisticated peel discoloration and pitting of peel, severe scalding, and an uneven ripening pattern that substantially stimulates the decay incidence in papaya [8]. Considerable research work has been performed to improve papaya shelf life and satisfy the consumer. Multiple postharvest preservation approaches such as exogenous melatonin [9], salicylic acid [10], calcium chloride [11], kojic acid [12], hot water [13,14], edible coatings [3,15,16,17], gamma irradiation [18], and modified atmosphere packaging and methyl jasmonate [19] were applied to improve the quality and the storability of papaya.

Methyl jasmonate (MeJA) is a phytohormone endogenously produced in higher plants and has been long used as a practical approach to maintain the shelf life and quality of many horticultural products because of its numerous physiological activities [20]. The exogenous MeJA treatment regulates the plant’s development processes and resistance against biotic and abiotic stress, promotes host defense resistance, and modulates fruit ripening and quality change [21]. MeJA could activate the antioxidant system that facilitated the elimination of reactive oxygen species and mitigated oxidative stress during postharvest storage of horticultural products [22,23]. Several studies reported that MeJA stimulated the accumulation of secondary metabolites and antioxidant activities that induced resistance to various postharvest disease in various fresh products and reduced the postharvest processing’s negative impact on quality and shelf life [24,25]. Exogenous MeJA application substantially prolonged storage life and reduced the fruit quality deterioration by improving membrane integrity and scavenging abilities of reactive oxygen species in strawberries [26,27] and grapes [28], and improved lipid metabolism in tomatoes [29].

Exogenous MeJA maintained the nutritional composition of loquats [30], Chinese bayberries, and grapes [31,32] during low-temperature storage. In addition, MeJA application has been reported to improve quality traits of fresh produce by activating the antioxidant system and reducing the chilling injury in plums and red raspberry fruits throughout postharvest storage [33,34]. Furthermore, MeJA reduced chilling symptoms by increasing the activity of antioxidant enzymes in tomato berries [35,36], peaches [37], lemons [38], and bell peppers [39] stored at low temperature. Similarly, Rehman et al. [40] demonstrated that MeJA-treated Valencia orange (Midknight) fruits showed markedly lower chilling injury symptoms during cold storage. Furthermore, MeJA-treated carambola fruits markedly prevented chilling injuries and exhibited notable increase in ascorbic acid content and synthesis of protective molecules which enhanced quality traits by improving shelf life during long storage [41]. Similarly, MeJA and salicylic acid treatment enhanced the chilling tolerance and improved the total phenolics and oxidative phenolic enzymes activities in lemon fruits during cold storage [42]. Furthermore, exogenous application of MeJA significantly decreased internal bruising, delayed senescence, and retained sensorial quality and, consequently, the shelf life of eggplants [43]. Moreover, González-Aguilar et al. [19] reported that postharvest MeJA application significantly inhibited the fungal decay, chilling development, and firmness deterioration of papaya fruits stored for 14–32 days at 10 °C and 4 days’ shelf life at 20 °C.

Exogenous MeJA applications have been widely used to retain and maintain the shelf life and quality of fresh fruits and vegetables. Keeping in mind the above report regarding the beneficial outcomes of MeJA application in attenuation of chilling injury, resistance against biotic and abiotic stress, enhancement of antioxidant activities, and enhancement of shelf life of horticultural commodities, we carried out our studies. However, the previous literature is limited concerning the efficacy of postharvest MeJA application on quality traits and shelf-life preservation of papaya fruits. In the current investigation, evaluations were carried out on the efficacy of MeJA to preserve the postharvest shelf life of papaya fruits under storage at 10 °C. The objective of the study was to assess the effectiveness of MeJA on attenuation of chilling injury, regulation of antioxidants enzymes, and preservation of quality attributes of papaya under low-temperature storage.

## 2. Materials and Methods

### 2.1. Plant Material and Sampling

Papaya (*Carica papaya* ‘Red lady’) fruits that were identical in color, size, and shape and free from any apparent disease were picked at the color break stage from a commercial papaya orchard located in Faisalabad, Punjab, Pakistan. In less than two hours, papaya fruits were wrapped in cardboard boxes and delivered to the laboratory. The papaya fruits were again selected in the lab, and those that were devoid of bruises, defects, and cracks were chosen for further tests. The selected fruits were rinsed with tap water, then sterilized for 2 min using a solution of 0.1% sodium hypochlorite, and then air-dried at room temperature (25 ± 2 °C) for nearly 40 min. The surface-sanitized fruits were randomly divided into two lots consisting of 150 papaya fruits each, with one lot being dipped in pure water (control) and the second lot being dipped in 1 mM methyl jasmonate (Sigma-Aldrich Company, USA) for 10 min, with each lot having three replicates of 50 fruits per treatment. We kept separate 15 papaya fruits (out of 150 papaya fruits) for weight loss assessment from each treatment. We previously performed a preliminary test on papaya fruit using four different concentrations of MeJA (0.5, 1, 1.5, and 2. mM). On the basis of the output of this experiment, the concentration of 1 mM MeJA was kept for future research. All papaya fruits were then air-dried and kept at 10 ± 2 °C and RH of 95% throughout 28 days. Fruit samples (9 fruits per replicate from a lot of remaining 135 fruits) were collected following MeJA treatment at different intervals (0, 7, 14, 21, and 28) across the storage duration to assess the physiological fruit weight loss (PWL), fruit decay, chilling injuries index, accumulation of malondialdehyde and hydrogen peroxide, total phenolics content, ascorbic acid content, the antioxidant activity (DPPH assay), soluble solids contents, titratable acidity, ripening index, pH, and the activity of antioxidant enzyme, as well as sensorial attributes.

### 2.2. Measurement of Physiological Weight Loss, Disease Incidence, and Chilling Injury Index of Papaya Fruit

For the analysis of PWL, papaya fruits (5 fruits per replicate) were weighed on the initial day (0 d) and at each storage time interval by using an electronic balance (EK-600H, Japan). Fruit PWL was determined by calculating variation between fresh and stored fruit weight and expressed as a percentage.

(1) $$Fruit\;weight\;loss\;(\%)=\left(Initial\;weight-Final\;weight\right)÷Initial\;weight\times 100$$

Fruit decay was monitored by counting the rotted fruit number (fruit showing 2–3 spots caused by pathogens) from the total number of fruits at each sampling day of storage time and was expressed as a percentage.

(2) $$Disease\;Incidence\left(\%\right)=Number\;of\;decayed\;fruit÷Total\;number\;of\;fruit\times 100$$

Chilling injury in papaya was estimated based on Nian et al. [7] by using a hedonic scale consisting of five points. Papaya fruit showing chilling symptoms (peel pitting and peel discoloration) was evaluated in terms of score as 1 (no chilling symptoms), 2 (1–25%), 3 (26–50%), 4 (51–75%), or 5 (above 75%). The chilling index was expressed as a percentage (%) using the calculation below:

(3) $$Chill\;ingindex\;(\%)=\frac{Chilling\;injury\;score\times Number\;of\;fruit\;showing\;chilling\;sign}{Total\;number\;of\;fruits}$$

### 2.3. Measurement of Malondialdehyde and Hydrogen Peroxide Accumulation of Papaya Fruit

The accumulation of malondialdehyde (MDA) was performed as previously reported by Hodges et al. [44], with minor differences. Papaya pulp fraction (1 g) was macerated in trichloroacetic acid (10 mL of 1%) solution and then centrifuged for approximately 10 min at 12,000× *g*. The upper layer was separated and used as a content assay. The collected supernatant (0.5 mL) was stirred with a solution of (0.2 mL of 0.5%) thiobarbituric acid and then heated at 96 °C for 15 min and finally cooled. The mixture was then centrifuged at 15,000× *g* for 5 min, and the supernatant was monitored at 532 nm, 600 nm, and 450 nm. MDA concentration was estimated as nmol g^−1^ FW using the following formula.

(4) $$MDA\;accumulation\left(\frac{nmol}{g}\right)=[\left(A532-A600\right)-0.56\times A450]$$

For the hydrogen peroxide (H_2_O_2_) accumulation experiment, a sample of (1 g) papaya flesh tissue was thoroughly homogenized with 5 mL of 1% trichloroacetic acid and centrifuged at 10,000× *g* for 20 min. Following that, supernatant (0.1 mL) was reacted with potassium iodide (0.1 mL of 1 M) and phosphate extraction buffer (0.1 mL of 100 mM, pH 7.0), and the solution’s absorbance was measured at 390 nm. The H_2_O_2_ concentration was estimated as mmol kg^−1^ fresh weight [9].

### 2.4. Measurement of Total Phenolic Content, 2,2 Diphenyl-1-picrylhydrazyl (DPPH) Radical Scavenging Activity, and Ascorbic Acid Content of Papaya Fruit

The total phenolic content of papaya juice was determined using the method proposed by Hanif et al. [10]. A sample of 1 g of papaya tissue was homogenized in a 5 mL extraction solution consisting of methanol, acetone, and HCl in a 90:8:2 ratio. Following a 10 min centrifugation at 15,000× *g*, the supernatant (0.5 mL) was treated with Folin–Ciocalteu and sodium bicarbonate (3 mL of 700 mM). The absorbance of the solution was measured at 765 nm. The data were given in milligrams of gallic acid equivalent per 100 g of fresh weight (mg 100^−1^ g FW).

The radical scavenging activity of DPPH was performed as outlined in Wang et al. [9]. In brief, a sample of (1 g) papaya flesh was thoroughly homogenized in ethanol (10 mL of 50%) and centrifuged under 4 °C during 15 min at 12,000 g. The supernatant (0.5 mL) was then reacted with the DPPH solution (0.2 mL) and kept in the dark at 25.2 °C for 30 min. The solution absorbance was measured at 517 nm, and the antioxidant activity was calculated as [(Ab − As)/Ab] 100, where Ab is the absorbance reading of the blank and As is the absorbance reading of the sample.

The papaya ascorbic acid content was determined as outlined in Ruck [45]. A papaya juice sample (10 mL) was extracted and placed into a 100 mL volumetric flask, then oxalic acid (90 mL at 0.4% concentration) was added to bring the volume up to 100 mL. Following that, the solution was filtered, and the filtrate (5 mL) was titrated against the 2, 6 dichlorophenol-indophenol dye until the endpoint pink color development that lasted at least 15 s. The results were given in milligrams per 100 g of fresh weight.

### 2.5. Measurement of Soluble Solid Content, Titratable Acidity, Ripening Index, and pH of Papaya Fruit

The content of soluble solid was determined with a digital refractometer (RX 5000, Atago, Japan). For this purpose, papaya fruit was peeled off and crushed in a juice blender. Papaya juice was filtered with Whatman filter paper. A drop from filtered papaya juice was employed to the cleaned prism of the refractometer, and the reading was noted through the scale at room temperature. The prism was properly cleaned with distilled water before and after sample reading. Total soluble solids were represented as °Brix (RX 5000, Atago, Japan).

Papaya fruit’s titratable acidity was assessed as highlighted by Hortwitz [46]. To perform this task, 5 mL fresh juice and 25 mL distilled water together with 2 to 3 drops of phenolphthalein indicator were put into a 100 mL conical flask. Subsequently, the solution was titrated with sodium hydroxide solution NaOH (0.1 N) until pink color was obtained for at least 15 s. The final reading was generated by the following equation, and TA was expressed as a percentage of citric acid (%).

(5) $$TA\;(\%)=\left(0.1\;N\;NaOH\times 0.0064\right)÷5\;mL\;juice\times 100$$

The ripening index of the papaya fruit was determined by dividing the SSC value by TA, while pH was measured with a digital pH meter.

### 2.6. Estimation of Sugars of Papaya Fruit

The sugar contents of papaya juice were determined by Fehling’s solution titration method, previously proposed by Hortwitz [46]. Precisely, 15 mL potassium oxalate solution and 30 mL of lead acetate were mixed with 5 mL freshly extracted juice of papaya in a 250 mL measuring flask. Following that, distilled water was added until 250 mL. The obtained solution was filtered using Whatman filter paper, and the filtrate was used for further sugar analysis. Finally, readings were described in percentage by the following formula.

(6) $$Total\;sugars\left(\%\right)=25\times (\frac{X}{Y})$$

(7) $$Reducing\;sugars\left(\%\right)=6.25\times (\frac{X}{Y})$$

where:

X = standard sugar value used during titration.
Y = value of filtrate used during titration.

(8) Nonreducing sugars=0.95×(Total sugar−Reducing sugar)

### 2.7. Determination of Antioxidative Enzyme Activities of Papaya Fruit

The modified technique proposed by Wang et al. [9] was used to estimate antioxidative enzymes. Enzyme extract was made by homogenizing 1 g frozen pulp tissue in 2 mL of 50 mM sodium phosphate buffer (pH 7.0) to measure the activity of superoxide dismutase (SOD), peroxidase (POD), and catalase (CAT). The mixture was then centrifuged at 12,000× *g* for 10 min at 4 °C, and the supernatant was separated and used for enzyme assays.

The SOD enzyme activity was determined as reported in Štajner and Popović [47]. In brief, the reaction solution was composed of 0.2 mL phosphate buffer (50 mM, pH 5), 0.2 mL 22 mM methionine, 0.1 mL 20 mM NBT, 0.2 mL 0.1 mM Titon-X, and 0.8 mL distilled water and incubated under UV lamps in the dark for 20 min. The reaction solution was then treated with 0.1 mL of 0.6 mM riboflavin. Following that, 0.1 mL of supernatant was reacted with 0.1 mL of freshly prepared reaction solution, and the mixture’s absorbance was measured at 560 nm and reported as U/mg protein. One unit of SOD activity was defined as the 50% inhibition rate of nitro blue tetrazolium per min.

The method of Wang et al. [9] was used for determining papaya POD. Simply, a reaction mixture (0.1 mL) of guaiacol (20 mM), hydrogen peroxide (40 mM), and sodium phosphate buffer (0.8 mL, pH 5) was reacted with enzyme extract (0.1 mL), and absorbance was measured at 470 nm and expressed as U/mg protein. The change absorbance at 470 nm during 1 min is defined as one unit of POD activity. CAT activity was determined following the procedure of Wang et al. [9]. The enzyme extract (0.1 mL) was reacted with hydrogen peroxide (0.1 mL of 20 mM), and the solution’s absorbance was measured at 470 nm. The activity of CAT was expressed as U/mg, and the quantity of enzyme leading to 0.1 min^−1^ absorbance change was considered as one unit. The protein content was assayed according to the method reported by Bradford [48]. Briefly, enzyme extract (0.1 mL) was reacted with Bradford reagent (0.16 mL), and absorbance was measured at 595 nm.

### 2.8. Determination of Sensory Attributes of Papaya Fruit

Sensory qualities of different papaya fruits were evaluated following the procedure of Lawless and Heymann [49]. Papaya fruits were evaluated for their taste, aroma, sweetness, and general acceptance by using a 9-point hedonic scale. For sensory rating, a panel of well-experienced judges (of all genders and ages) were hired. Papaya fruit slices were provided to the judges for examination on properly labelled disposable plates. All judges were asked to offer a score ranging from 1 to 9, with 1 indicating dislike strongly, 5 indicating neither like nor dislike, and 9 indicating like very much.

### 2.9. Statistics

All statistical analyses of our experiment were executed according to a complete randomized design by using statistic 8.1 software. The data were analyzed by factorial arrangement under a two-way variance assessment (ANOVA) and reported as mean ± standard error (SE). The least significant difference (LSD) test at *p* ≤ 0.05 level was employed to highlight significant differences between applied treatments. Each treatment consisted of three individual biological replications.

## 3. Results

### 3.1. MeJA Effects on Physiological Weight Loss, Disease Incidence, and Chilling Injury

**Figure 1:**
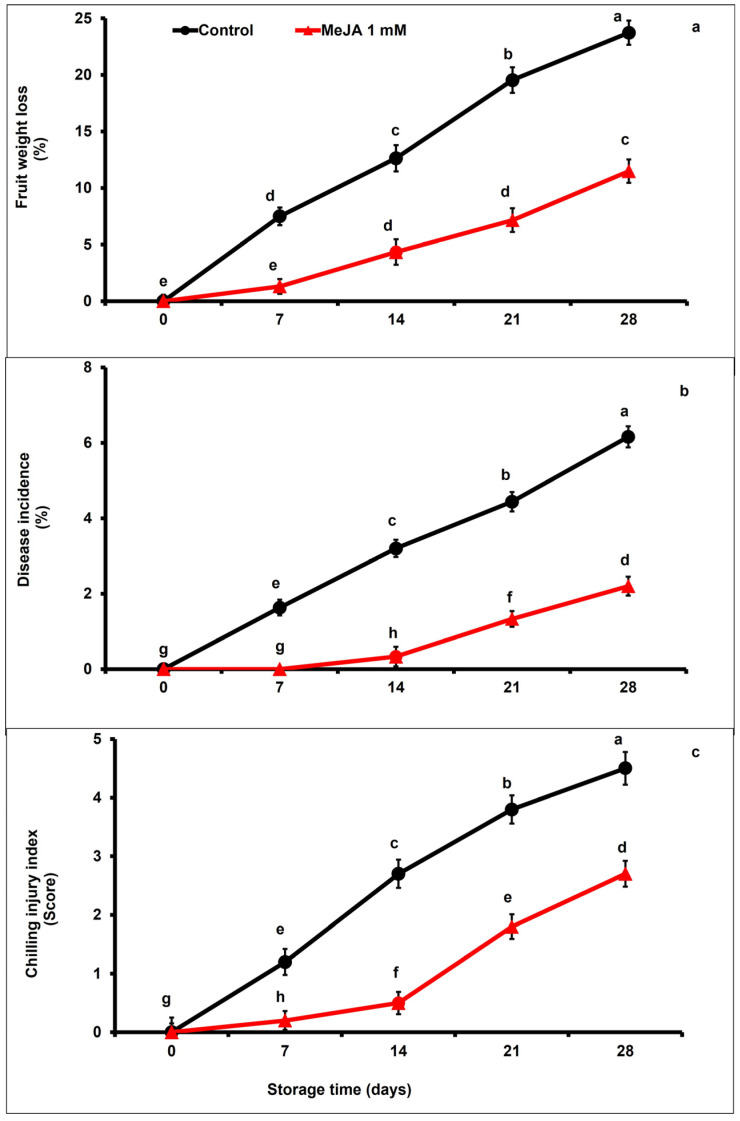
The variation affecting physiological weight loss (**a**), disease incidence (**b**), and chilling injury (**c**) of papaya fruits following MeJA treatment. The data were acquired using three replicates, and the vertical bars represent the standard error of means. According to the least significant difference test (*p* < 0.05), mean values with different letters show statistically significant differences, whereas mean values with the same letters show no statistically significant difference.

The PWL affecting the papaya fruits and increased gradually with the storage period, regardless of MeJA treatment and control group (*p* < 0.05; Figure 1a). However, MeJA-treated papaya fruits exhibited significantly lower physiological fruit weight loss in contrast to the untreated fruits across the storage period (*p* < 0.05). Furthermore, fruits subjected to MeJA treatment were 2.06 times lower in PWL than untreated fruits, as recorded at the end of the storage period (Figure 1a).

The incidence of disease increased markedly across the entire cold storage duration in both MeJA-treated and control papaya fruits (Figure 1b). However, the fruit decay generally remained lower during the first 14 days in MeJA-treated papaya fruits with respect to untreated fruits (*p* < 0.05). Moreover, MeJA-treated papaya fruits showed a few decay symptoms until day 14 (9-fold lower), and then showed a 3.3-fold lower decay incidence on day 21 of storage than untreated fruits, respectively. At the end of the cold storage period, disease occurrence in MeJA-treated fruits was 2.8 times lower with respect to the untreated fruits (*p* < 0.05; Figure 1b).

Regardless of applied treatment, chilling injury in papaya fruits increased throughout the storage period (Figure 1c). However, MeJA drastically delayed the increase in chilling injury in comparison to the control papaya fruits. MeJA-treated papaya fruits significantly (*p* < 0.05) showed 2.11-fold and 1.67-fold lower chilling injury compared to untreated fruits at day 21 and day 28 of storage, respectively (Figure 1c).

### 3.2. MeJA Effects on Malondialdehyde and Hydrogen Peroxide Accumulation

**Figure 2:**
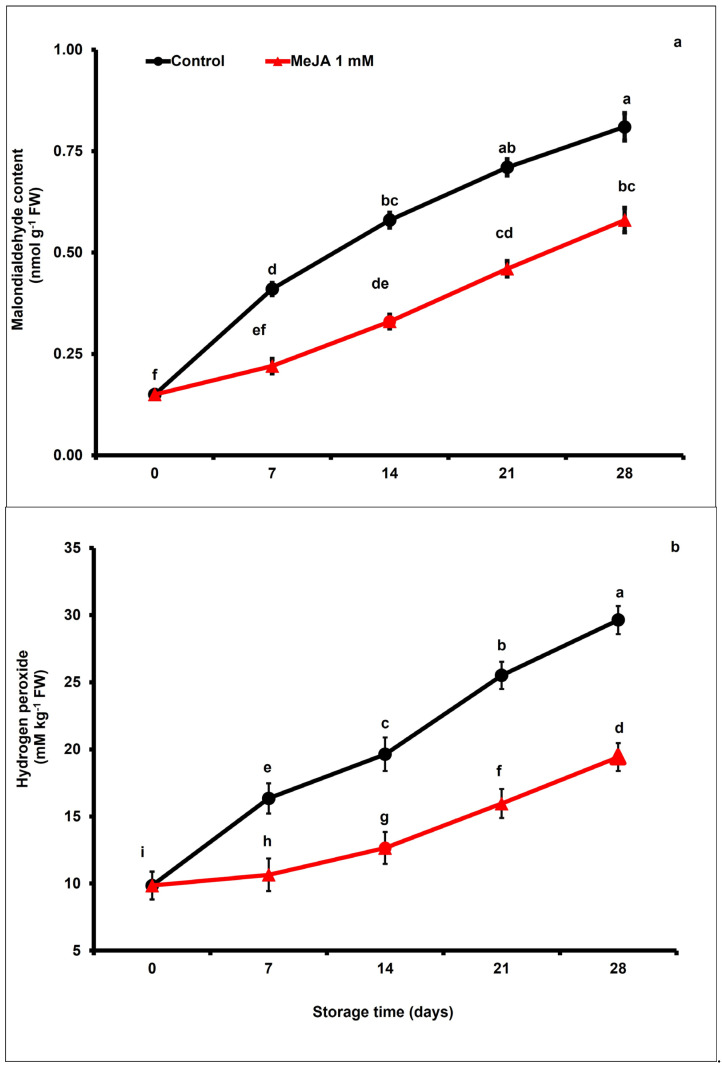
The variation affecting malondialdehyde (**a**), and hydrogen peroxide accumulation (**b**) in papaya fruits following MeJA treatment. The data were acquired using three replicates, and the vertical bars represent the standard error of means. According to the least significant difference test (*p* < 0.05), mean values with the same letters show no statistically significant difference.

Malondialdehyde (MDA) content in papaya fruits gradually increased during postharvest storage. Nevertheless, exogenous MeJA treatment markedly suppressed the increase in malondialdehyde content with respect to control fruits. After day 28 of storage, the concentration of malondialdehyde was noticeably 1.39-fold lower in papaya fruits treated with MeJA than the control fruits (*p* < 0.05; Figure 2a).

The hydrogen peroxide (H_2_O_2_) production rate significantly increased in both MeJA-treated and untreated papaya fruits during the storage period (*p* < 0.05; Figure 2b). Nevertheless, a marked increase in H_2_O_2_ content was noticed in the control compared to the MeJA-treated papaya fruits. On day 28 of the storage period, hydrogen peroxide accumulation in MeJA-treated papaya fruits was approximately 1.52-fold lower than in untreated fruits (Figure 2b).

### 3.3. MeJA Effects on Total Phenolic Content, Ascorbic Acid Content, and 2,2-Diphenyl-1-picrylhydrazyl (DPPH) Radical Scavenging Activity

**Figure 3:**
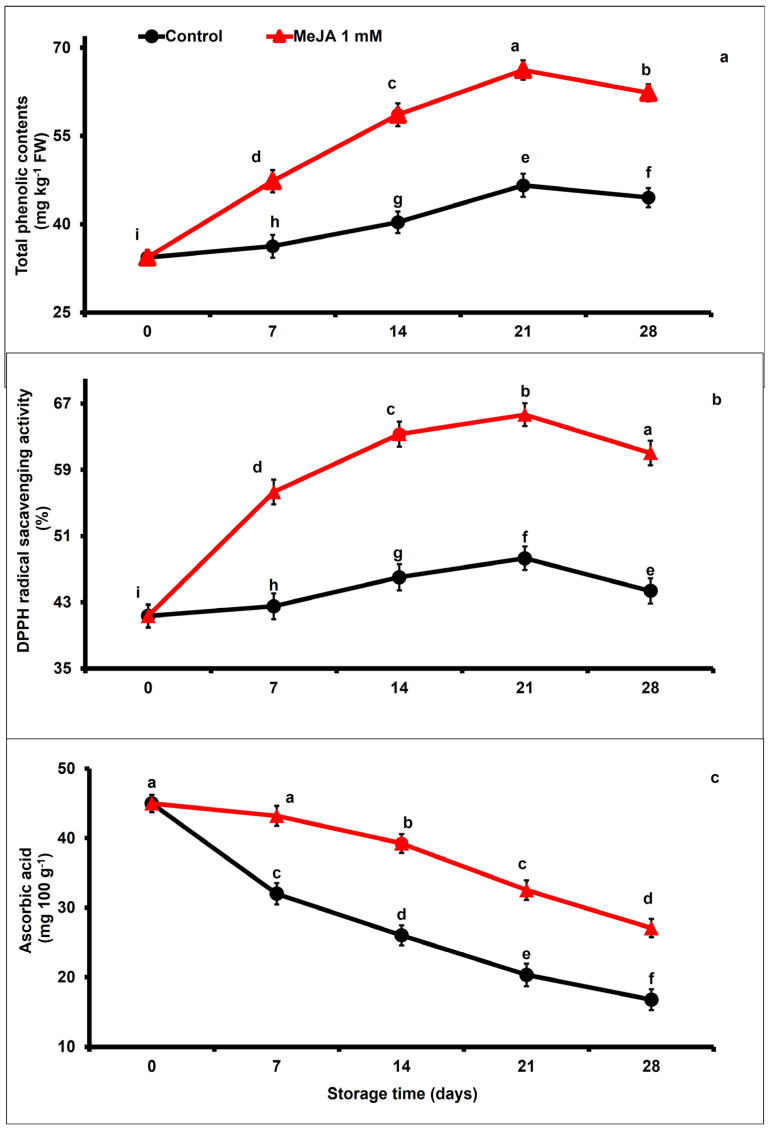
The variation affecting total phenolic contents (**a**), DPPH radical scavenging activity (**b**), and ascorbic acid content (**c**) in papaya fruits following MeJA treatment. The data were acquired using three replicates, and the vertical bars represent the standard error of means. According to the least significant difference test (*p* < 0.05), mean values with different letters show statistically significant differences, whereas mean values with the same letters show no statistically significant difference.

The levels of total phenolic content in papaya fruits gradually increased between day 7 and day 21, and then decreased until the end of the storage period, regardless of the applied treatment (Figure 3a). However, MeJA-treated papaya fruits retained substantially higher total phenolics with respect to control fruits. At the end of the storage (day 28), total phenolic content was 1.40-fold higher in MeJA-treated than in untreated papaya fruits (*p* < 0.05; Figure 3a).

The antioxidant activity gradually increased and then decreased in stored papaya regardless of treatments (Figure 3b). Nevertheless, MeJA-treated papaya fruits exhibited 1.19-fold higher DPPH scavenging potential after 3 weeks of storage (day 21) compared to untreated papaya fruits, whereas on day 28, DDPH scavenging potential decreased but remained 1.37-fold higher in MeJA-treated than in untreated papaya fruits (Figure 3b).

Ascorbic acid content substantially decreased in papaya fruits during the extension of storage duration. Nevertheless, MeJA application substantially (*p* < 0.05) retained higher levels of ascorbic acid concentration as compared with the untreated group (Figure 3c). At the end of the storage period (day 28), ascorbic acid content was 1.61-fold higher in MeJA-treated than in untreated papaya fruits.

### 3.4. MeJA Effects on Soluble Solid Content, Titratable Acidity, Ripening Index, and pH

**Figure 4:**
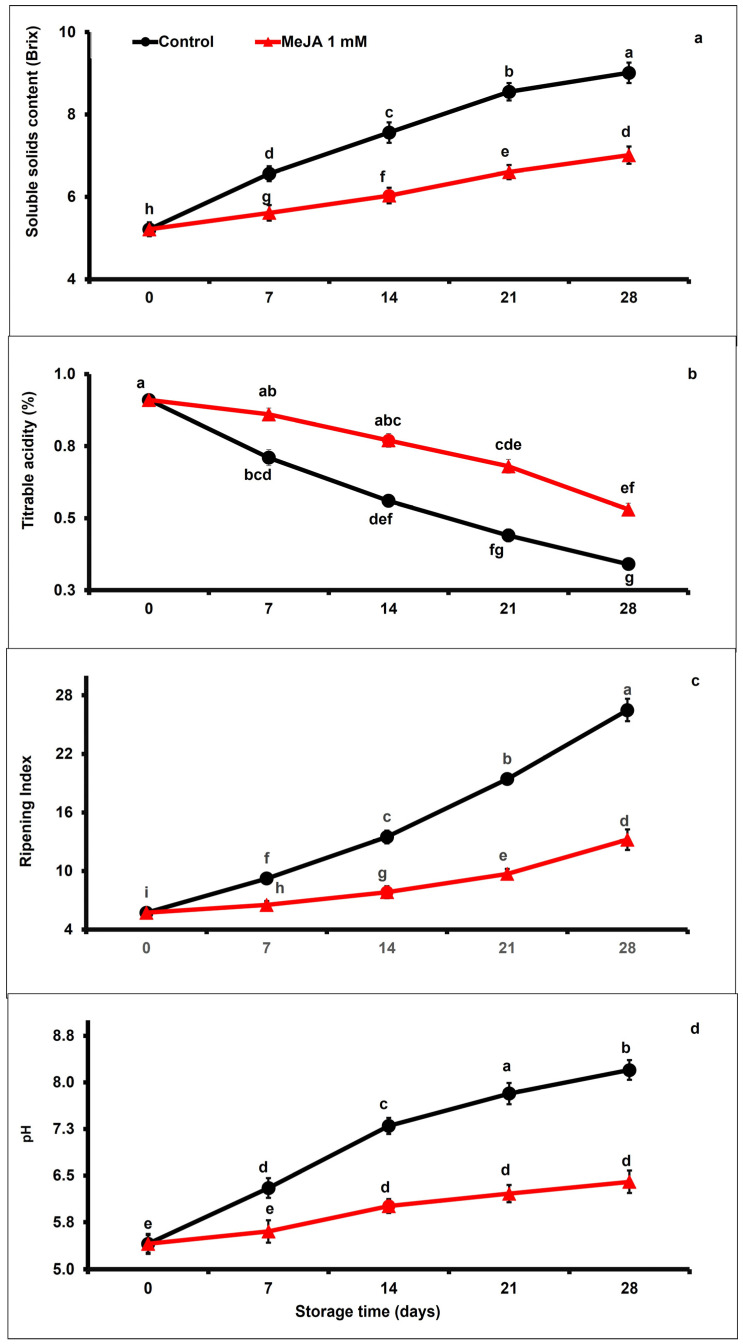
The variation affecting soluble solid contents (**a**), titratable acidity (**b**), ripening index (**c**), and pH (**d**) in papaya fruits following MeJA treatment. The data were acquired using three replicates, and the vertical bars represent the standard error of means. According to the least significant difference test (*p* < 0.05), mean values with different letters show statistically significant differences, whereas mean values with the same letters show no statistically significant difference.

The content of the soluble solid increased moderately in fruit during the extension of storage. However, MeJA treatment decreased the soluble solid content more significantly than in untreated fruits throughout the storage period (Figure 4a). MeJA treatment markedly suppressed soluble solids content (0.77-fold) as compared with untreated fruits up to day 28 of storage (*p* < 0.05; Figure 4a).

Disregarding the treatment, titratable acidity values of papaya fruit markedly (*p* < 0.05) decreased under storage (Figure 4b). However, MeJA treatment significantly increased titratable acidity when compared to control papaya fruits. Titratable acidity was 1.55-fold higher in MeJA-treated than in control fruits at the end of the storage period.

Regardless of treatments, the ripening index increased moderately with advancing storage (Figure 4c). Nevertheless, exogenous MeJA application significantly (*p* < 0.05) decreased ripening index values as compared with untreated papaya fruits. After day 28 of storage, the ripening index was remarkably 0.49-fold lower in MeJA-treated compared to untreated papaya fruits (Figure 4c). Likewise, pH gradually increased between day 7 and day 28 of storage, regardless of the applied treatment. Furthermore, 0.78-fold higher pH values were recorded in untreated than in MeJA-treated papaya fruits.

### 3.5. MeJA Effects on Total Sugar, Reducing Sugar, and Nonreducing Sugar

Disregarding the applied treatments, the content of total sugars as well as reducing and nonreducing sugars substantially increased in papaya fruits throughout the storage period (*p* < 0.05; Table 1). Nevertheless, exogenous MeJA application considerably decreased their content across the storage as compared with untreated papaya fruits. After day 28, total, reducing, and nonreducing sugars noticeably were 0.61-fold, 0.71-fold, and 0.49-fold lower, respectively, in MeJA-treated than in untreated papaya fruits.

### 3.6. MeJA Effects on Activities of Antioxidant Enzyme

**Figure 5:**
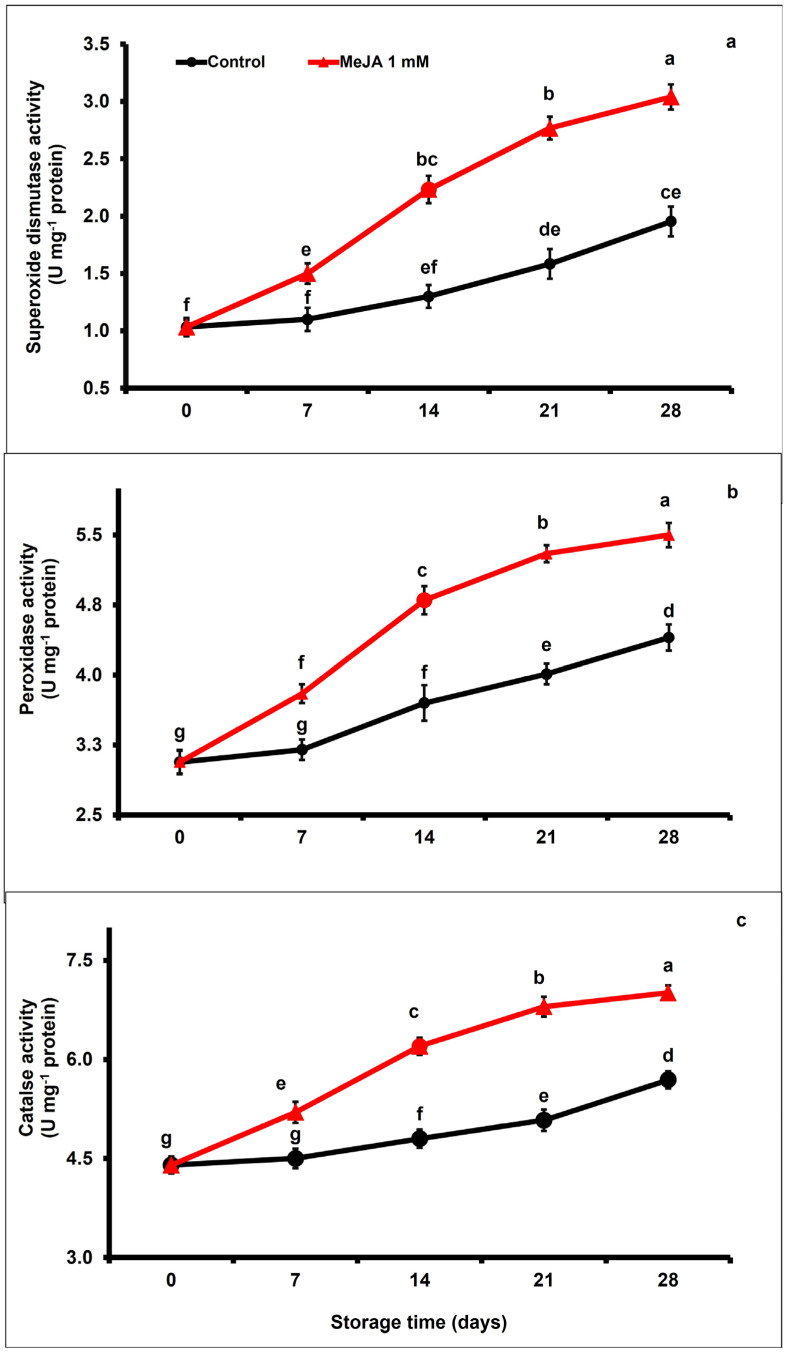
The variation affecting superoxide dismutase (**a**), peroxidase (**b**), and catalase (**c**) in papaya fruits following MeJA treatment. The data were acquired using three replicates, and the vertical bars represent the standard error of means. According to the least significant difference test (*p* < 0.05), mean values with different letters show statistically significant differences, whereas mean values with the same letters show no statistically significant difference.

Regardless of the applied treatment, SOD, POD, and CAT enzymatic activity of papaya fruits markedly increased (*p* < 0.05; Figure 5a–c) between day 7 and day 21 and decreased afterwards at the end of the storage period. However, exogenous application of MeJA increased SOD, POD, and CAT enzymatic activity across storage with respect to control papaya fruits. At the end of the storage period (day 28), SOD, POD, and CAT activities were 1.55-fold, 1.22-fold, and 1.23-fold higher, respectively, in MeJA-treated than in untreated papaya fruits.

### 3.7. MeJA Effects on Sensory Attributes

**Figure 6:**
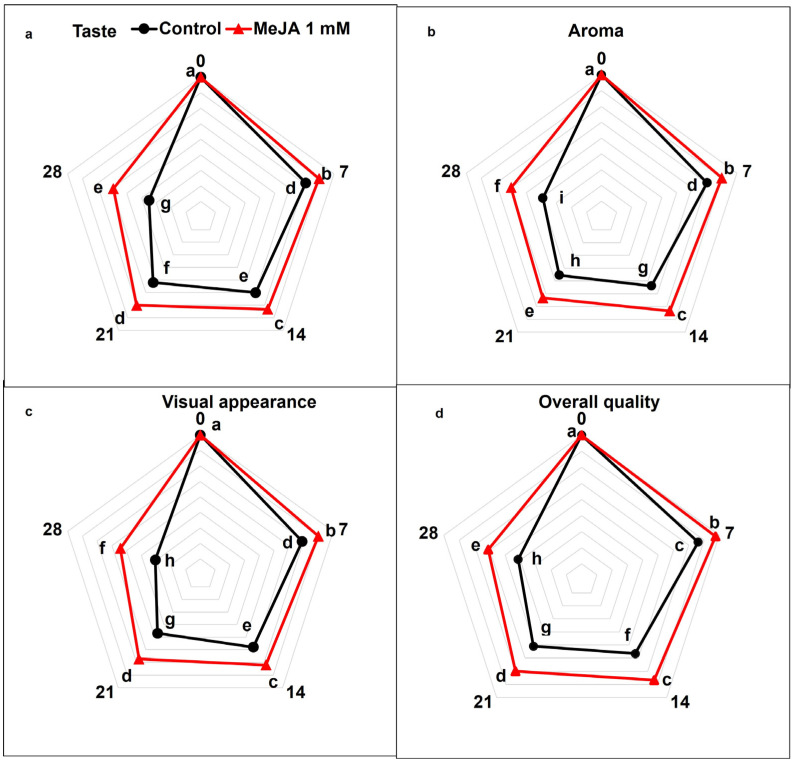
The variation affecting taste (**a**), aroma (**b**), visual appearance (**c**), and overall acceptability (**d**) in papaya fruits following MeJA treatment. The data were acquired using three replicates, and the vertical bars represent the standard error of means. According to the least significant difference test (*p* < 0.05), mean values with different letters show statistically significant differences, whereas mean values with the same letters show no statistically significant difference.

The recorded scores for taste, flavor, and aroma as well as general acceptability of papaya fruits considerably decreased in all treatments between day 7 and day 28 of storage (*p* < 0.05; Figure 6a–d). However, taste, aroma, visual appearance, and overall acceptability was markedly lower in untreated control papaya fruits than in MeJA-treated papaya fruits. On day 28 of cold storage, taste, aroma, visual appearance, and overall acceptability scores of MeJA were 1.68-fold, 1.54-fold, 1.77-fold, and 1.46-fold higher than control papaya fruit treatments (*p* < 0.05; Figure 6a–d).

## 4. Discussion

Papaya fruit quality can be assessed through the measurement of weight loss during postharvest storage, which is a vital indicator of fresh fruit quality [9,10]. Fruits and vegetables are more affected by PWL after harvesting due to the high rate of respiration, transpiration, and physiological metabolism [50]. The loss of fresh weight causes severe wrinkling of the fruit surface, which leads to high postharvest losses with inferior quality in fruit crops during their transportation and storage. In our experiment, the PWL of papaya fruits markedly increased with advances in storage duration. Nevertheless, MeJA-treated papaya fruits significantly reduced PWL as compared with the control group (Figure 1a), which might be ascribed to deferred respiration and transpiration. The presented data are in agreement with previous findings on radishes [51], ‘Arrayana’ mandarins [52], apricots [53], and kinnows [54], where MeJA application substantially reduced PWL by reducing the transpiration and maintaining membrane integrity. Furthermore, Liao et al. [38] noticed a decrease in PWL and chilling injury symptoms in lemon fruit during storage following pre- and postharvest MeJA application ascribed to delayed metabolic activities and senescence.

Postharvest disease development badly influences the fruit’s appearance due to the presence of sunken lesions, resulting in fruit quality losses and considerable decline in the market price of fresh produce [55]. Recent studies have demonstrated that MeJA application significantly decreases disease incidence and maintains postharvest quality of fruit such as kiwifruit, cherry, and Chinese bayberries [31,56,57]. In this study, the disease incidence in papaya fruit was moderately increased with advances in storage duration. MeJA-treated papaya fruits showed slight decay symptoms up to day 14 during cold storage with respect to untreated papaya fruits (Figure 1b). Comparable results were reported for kinnows [53], lemons [38], and banana fruits [58] during postharvest storage where exogenous MeJA treatment effectively reduced the disease incidence.

**Figure 7:**
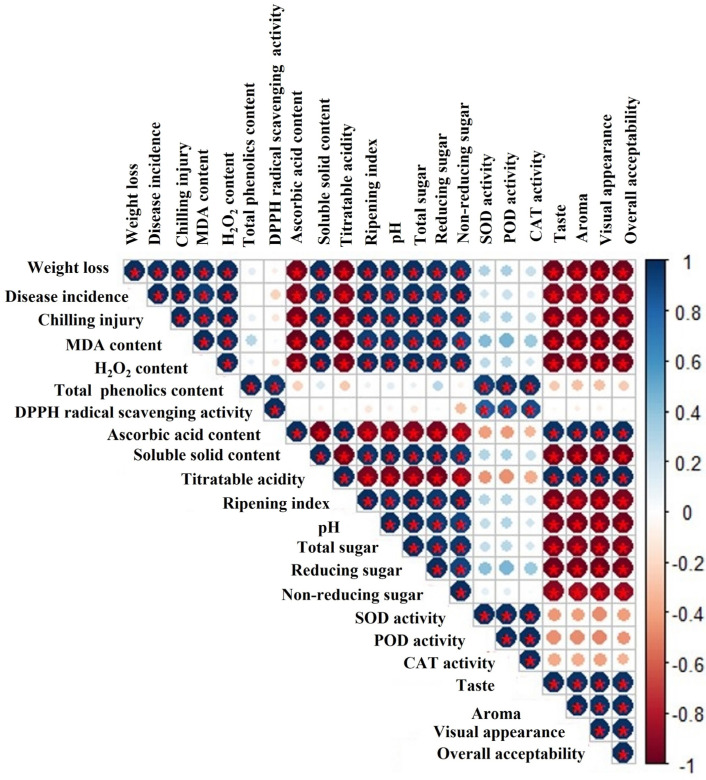
Pearson correlation coefficient heat map of various fruit quality attributes of MeJA-treated papaya fruit during low-temperature storage. The scale ranges from −1 to 1 (−1 = red, 1 = blue): red color represents negative correlation and blue indicate the positive correlation. The color gradient illustrates the intensity of the correlation. The symbol * denotes a statistically significant (*p* < 0.05) association.

Chilling injury badly influences the marketing and commercialization of sensitive fresh produce and is among the main postharvest issues. In general, chilling injury mainly happens due to loss of membrane firmness caused by consequences of oxidative stress. MeJA induces chilling-injury resistance by improving antioxidant enzyme activities. In our study, Pearson correlation analysis was conducted, and we noted a negative correlation between chilling injury and antioxidant enzymes (Figure 7). Cao et al. [59] reported that MeJA significantly reduced chilling incidence in loquat fruits by stimulating superoxide dismutase, ascorbate peroxidase, and catalase activates, while a lower chilling index in peach fruits was ascribed to increased peroxidase activity [60]. In the current study, MeJA markedly mitigated the chilling injury and maintained the membrane integrity, which might be due to improving antioxidant enzymes activates (Figure 1c). The obtained data are in harmony with various reports supporting that postharvest MeJA significantly alleviated chilling injury in bell peppers [39] guavas [61], mangos [62], loquats [30], Valencia oranges [40], and peaches [63].

Malondialdehyde (MDA) and hydrogen peroxide (H_2_O_2_) accumulation is generally associated with oxidative deterioration to the lipid membrane integrity and cold stress. Consequently, the lipid peroxidation level under MDA and H_2_O_2_ synthesis is tightly correlated with chilling injury levels and severity [64]. Moreover, a strong positive correlation of MDA and H_2_O_2_ with chilling injury was also observed during storage (Figure 7). Exogenous MeJA application can regulate antioxidant enzymes that are responsible for improving chilling resistance and protecting against membrane damage [63]. In our study, MeJA-treated papaya fruits exhibited lower levels of MDA and H_2_O_2_ than untreated fruits, which can be ascribed to higher contents of enzymatic and non-enzymatic antioxidants (Figure 2a,b). Similarly, Wang et al. [65] also showed that lower MDA and H_2_O_2_ accumulation in blueberries was directly correlated with a higher accumulation of non-enzymatic and enzymatic antioxidants. The obtained data are in harmony with Jin et al. [63], who stated that MeJA treatment inhibited MDA accumulation in peach fruits, which subsequently suppressed membrane damage and reduced chilling injury of peach fruits.

Total phenolic contents and DPPH scavenging potential are important bioactive compounds which are considered to be key health-promoting chemicals in papaya fruit [10]. In general, an increase in total phenolic content caused by MeJA treatment might be attributed to increased activity of the phenylalanine ammonia-lyase (PAL) enzyme, which is responsible for phenolic compound synthesis [61,66]. Moreover, MeJA significantly delayed the phenolic compound oxidation by reducing the activity polyphenol oxidase (PPO) enzyme [26]. In this study, papaya fruits treated using MeJA showed a remarkably higher accumulation of total phenolics than untreated control papaya fruits (Figure 3a,b). Similarly, pre- and postharvest MeJA treatment promoted total phenolic contents by increasing the enzyme activities of phenylalanine ammonia-lyase in regulating polyphenol synthesis [37,67,68,69,70,71]. Likewise, our results are supported by previous studies that concentrations of total phenolics and flavonoids increased with MeJA treatment in blueberries [50], pomegranates [72], and bayberries [66]. A previous study showed that higher total phenolic and antioxidant activity was maintained due to the amplification of phenylalanine ammonia-lyase activity following pre- and postharvest MeJA application in cherries [73]. The present results are in agreement with those of García-Pastor et al. [73], who noticed that pre- and postharvest MeJA-treated pomegranate fruits accumulated higher phenolics and anthocyanins until the end of the storage time. Despite this, retention of higher antioxidant activity in papaya fruits treated with MeJA is directly associated with higher TPC content, as revealed in Figure 7. In addition, Cao et al. [59] noticed that phenolics and anthocyanins hold a crucial capacity in maintaining higher radical scavenging activity. The obtained results are in harmony with findings suggesting that postharvest MeJA treatment significantly increased the radical scavenging activity in blood oranges [74], Chinese chives [75], and Chinese bayberries [66]. Likewise, it has been reported that higher total phenolic content may be responsible for enhanced DPPH radical scavenging activity in plants [28].

Ascorbic acid is an important health-promoting compound with high antioxidant potential to significantly remove reactive oxygen species and delays postharvest senescence [76]. The ascorbic acid levels are decreased under storage. In the present study, exogenous MeJA application managed to increase ascorbic acid accumulation compared to untreated papaya fruits across storage (Figure 3b). An increased level of ascorbic acid may be due to lower ascorbic acid oxidase activity, which stimulates ripening and senescence [77]. Postharvest application of individual or combined coatings of different chemicals maintained higher endogenous concentrations of ascorbic acid and reduced the oxidation process in apricot fruits during ambient storage [78]. In a similar way, MeJA application maintained higher levels of ascorbic acids in kinnows [54].

SSC is considered a major biochemical trait in the measurement of papaya fresh quality during storage [9]. The SSC concentration in fresh produce increases due to water loss and reduction in weight [79]. In the present study, exogenous application of MeJA decreased SSC values compared to the untreated control group (Figure 4a), due to lower metabolic activities and less weight reduction. Previous studies outlined that postharvest MeJA application significantly deferred the SSC in mangos [61], sweet cherries [73], strawberries [80], and apples [81].

Organic acids are usually present at higher concentrations in less ripe fruit and decrease through fruit ripening and postharvest senescence [77]. TA concentration decreases during storage due to the influence of metabolic changes in fruit or the utilization of organic acids in respiratory and postharvest senescence [82]. In the present study, the postharvest application of MeJA retarded the decrease in TA concentration as compared with the control papaya fruits (Figure 4b). A similar finding was reported that exogenous MeJA dipping showed higher TA content and delayed the utilization of organic acid, therefore preserving the marketable fruit quality of strawberries [80], kinnows [76], and sweet cherries [73]. However, MeJA applications showed no substantial changes in SSC and TA values in ‘Cripps Pink’ apples [69] and Delicious and ‘Golden Delicious’ apples [83], whereas MeJA applications increased SSC and decreased TA values in peaches [59], blackberries [84], and raspberries [85].

The ripening index (SSC: TA ratio) indicates the maturity indices of fruits for harvest time and fetches higher prices from the market. Previously, external MeJA application was reported to have ripening-retarding effects on peaches [86] and sweet cherries [73]. The lower ripening index depicted less consumption of organic acid and sugars substrate in different metabolic activities during the ripening and senescence process. Exogenous MeJA dipping exhibited a lower ripening index, which may retard the postharvest senescence in papaya fruits (Figure 4b).

Sugars are crucial to measuring sweetness in papaya, and lower concentrations of sugars represent long postharvest life during storage. Generally, the levels of reducing sugars and dry soluble extracts increase during storage of *Carica papaya* L. var Solo 8. Nevertheless, the pattern of change in total sugars exhibited an increasing trend initially followed by a slightly decreasing trend across storage (Table 1). This total sugars decrease is due to the increasing consumption of the saccharose due to climacteric respiration, as outlined in Yao et al. [24] using papaya cv. Solo 8. The authors also found that soluble sugars accumulation was mostly attained in on-vine fruits.

Plant tissues contain antioxidant enzymes (SOD, POD, and CAT) that combat reactive oxygens species. SOD converts superoxide anion to hydrogen peroxide, and CAT converts hydrogen peroxide to water and oxygen via various metabolic pathways [87]. In the presence of hydrogen peroxide, POD catalyzes different oxidation reactions [88]. In this work, the MeJA application conserved higher SOD, POD, and CAT activities than in the untreated control group (Figure 5a–c). Therefore, exogenous MeJA application can efficiently extend postharvest storage quality of papaya fruit. An increase in antioxidant enzyme activities (SOD, POD, and CAT) could enhance the DPPH scavenging potential of papaya fruit and maintain its quality attributes. Our results are in accordance with those reported by Sun et al. [89], who outlined that MeJA treatment improved POD and CAT enzyme activities by regulating the ROS production in banana fruits and in nonclimacteric eggplants [43]. Similarly, Chanjirakul et al. [90] noticed that exogenous MeJA application reduced the resistance to various diseases and enhanced the wide range of antioxidant enzyme activities in tomatoes during storage [29,35].

Sensory attributes represent an attraction factor for consumers and depend on sugar and acid composition [91]. In the present experiment, scores of sensory quality traits (taste, aroma, flavor, and overall acceptability) decreased during storage, but MeJA-treated papaya fruits exhibited higher scores of sensory quality traits than untreated fruits (Figure 6a–d). Overall, sensory traits decreased due to continuous changes in weight loss, decay, and various compounds such as volatiles, sugars, and organic acids under storage conditions [53,92]. Sensory quality is reduced due to disease incidence, weight loss, and decline in organic acid during storage, as revealed in Figure 7. It is suggested that MeJA application retards decay and reduces physiological changes in fruit. The obtained results are in accordance with previous studies focused on MeJA treatments and concluded positive outcomes on apricot and stone fruits’ sensory quality under storage [53,93].

## 5. Conclusions

In summary, our findings showed that exogenous MeJA application significantly mitigated chilling injury, conserved higher antioxidant activities, and maintained the quality attributes of papaya. Overall, MeJA-treated fruit exhibited lower physiological weight loss (PWL), disease incidence, and chilling injury than untreated fruits. Moreover, MeJA markedly inhibited malondialdehyde (MDA) and hydrogen peroxide (H_2_O_2_) synthesis and accumulation but stimulated the accumulation of phenolics and ascorbic acid content, and led to higher radical scavenging activity values compared to untreated papaya fruits. In addition, postharvest MeJA application clearly enhanced superoxide dismutase (SOD), peroxidase (POD), and catalase (CAT) enzyme activity along with acceptable biochemical and sensory quality. Therefore, postharvest MeJA application could be considered as a safe and suitable approach to mitigate disease development.

## Figures and Tables

**Table 1:** The variation affecting total sugar, reducing sugar, and nonreducing sugar in papaya following MeJA application under low-temperature storage.

Treatment	Storage Times (Days)				
Total sugar	0 d	7 d	14 d	21 d	28 d
Control	6.15 ± 0.11 h	8.54 ± 0.16 e	9.63 ± 0.21 c	12.7 ± 0.19 b	14.46 ± 0.16 a
MeJA 1 mM	6.15 ± 0.12 h	6.86 ± 0.17 g	7.54 ± 0.23 f	8.54 ± 0.17 e	8.84 ± 0.052 d
Reducing sugar					
Control	4.13 ± 0.21 h	6.21 ± 0.13 e	6.84 ± 0.16 c	8.07 ± 0.19 b	9.73 ± 0.17 a
MeJA 1 mM	4.13 ± 0.19 h	5.34 ± 0.16 g	5.51 ± 0.14 f	6.64 ± 0.21 d	6.95 ± 0.16 c
Nonreducing sugar					
Control	2.14 ± 0.04 f	3.01 ± 0.05 d	3.46 ± 0.06 c	4.13 ± 0.05 b	5.91 ± 0.04 a
MeJA 1 mM	2.14 ± 0.03 f	1.97 ± 0.04 g	2.01 ± 0.03 fg	2.48 ± 0.04 e	2.92 ± 0.03 d

The data were acquired using three replicates, and the standard error indicates the means of replication. Mean values with different letters show statistically significant differences, whereas mean values with the same letters show no statistically significant difference.

## Data Availability

The data presented in this study are available on request from the corresponding author.
